# The concept of entropic rectifier facing experiments

**DOI:** 10.1038/srep38966

**Published:** 2016-12-12

**Authors:** D. Lairez, M.-C. Clochard, J.-E. Wegrowe

**Affiliations:** 1 Laboratoire Léon Brillouin, CNRS, CEA, Université Paris-Saclay, 91191 Gif-sur-Yvette cedex, France; 2 Laboratoire des Solides Irradiés, École polytechnique, CNRS, CEA, Université Paris-Saclay, 91128 Palaiseau cedex, France

## Abstract

The transport of molecules in confined media is subject to entropic barriers. So theoretically, asymmetry of the confinement length may lead to molecular ratchets with entropy as the only driving force for the biased transport. We address experimentally this question by performing alternative ionic current measurements on electrolytes confined in neutral conical nanopores. In case anions and cations widely differ in size, we show that rectification of ionic current can be obtained that depends on ions size and cycle frequency, consistently with the entropic ratchet mechanism.

In fluids, due to random motion of molecules, their directed transport is ordinarily achieved by the mean of an external force field. Brownian motors and ratchets^1^,^2^,^3^ fulfill this task without net bias, not only despite random motion but thanks to it. Initially viewed as some thermodynamical curiosities for physicists^4^,^5^,^6^, these brownian machines have gained interest when they proven to be relevant in many biological systems, from muscle contraction^7^,^8^ to ionic pumps^9^, including voltage-gated channels^10^. Nowadays, advances in nanodevices fabrication stimulate bio-inspired realizations mostly concerning smart ions transport and pumping that likely promises the more challenging applications (e.g. keep in mind water desalination issues).

Ion pumps typically work with nanoporous membranes submitted to a periodic voltage, which is unbiased in average but allows the machines to fulfill the second law of thermodynamics^4^,^5^,^6^. Then, these machines are mostly founded on random motion of ions in a sawtooth landscape of potential that materializes the ratchet. All efforts to produce bio-inspired ionic current rectifiers lie in this materialization^11^. Until now, concrete realizations^12^,^13^,^14^,^15^,^16^,^17^,^18^ generate the ratchet by means of electrostatic charges on pore-wall, the asymmetry arises from a charge gradient or from the conical shape of nanopores that is felt by ions through electrostatic interactions^19^.

In this paper, we consider experimentally a more direct and universal way for any particles to feel the shape of nanopores that could come from entropic barriers^20^. The idea is the following. For ions with diffusion coefficient *D*, in solution at concentration *c*, moving in a potential *U*, the current *J* is the sum of brownian diffusion −*D*∇*c* and drift motion −*Dc*∇*U*/*kT*:


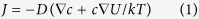


In confined systems, entropy changes are crucial and the thermodynamic potential to consider in Eq. 1 is more suitably the free energy *F* = *U* − *TS*. The substitution of *U* by *F* introduces the entropic forces *T*∇*S*. Although these forces do not originate from the microscopic level but rather emerge from the statistics of the entire macroscopic population of particles, they are palpable and have proven to be of great importance in many systems (see e.g. the elasticity of polymer chains^21^ or the depletion force in colloids^22^). In a narrow channel, if the transverse equilibrium is rapidly reached, the problem can be treated in 1D using the longitudinal probability density *p*(*x*) = *c*(*x*)*A*(*x*), with *A*(*x*) the cross-section area. In ideal solutions, the number of microstates for one given ion is Ω ∝ *A*(*x*)/*a*, with *a* the area unit. The corresponding entropy is *k*ln(*A*(*x*)/*a*) and Eq. 1 without external potential leads to the Fick-Jacobs equation^23^:


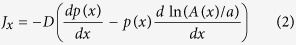


Eq. 2 is still insufficient to rectify ionic current because charge carriers are dual and the entropic contribution to the free energy is repulsive for both species (contrary to the potential due to pore-wall charge). Thus when transport is facilitated for anions it is impeded for cations and vice versa and the free energy potential remains symmetrical in average. To break the symmetry, the missing piece is related to the depletion layer^22^: the center of mass of ions cannot approach the wall to distance smaller than their radius *r*, so the accessible area is rather 
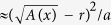
24,25. Then, if the pore section is sawtooth, one gets size-dependent entropic barriers that can be used to split ions regarding to their size^25^. Also, if this idea is correct, thus it should be possible to rectify the ionic current passing through geometrically asymmetric nanopores in case anions and cations widely differ in size (Fig. 1). This is what we experimentally address and report in this paper.

Our experiments consist in ionic current measurements as a function of the applied sinusoidal voltage (cyclic voltammetry) through conical nanopores (track-etched membranes similar to those used in refs 12 and 13) using polyanions as charge carrier (polyacrylic acids, PAA). We show that at pH conditions insuring the electric neutrality of pore-wall, a clear ionic current rectification can be still obtained that depends on polyanions size and cycle frequency.

## Experimental details

**Figure 2:**
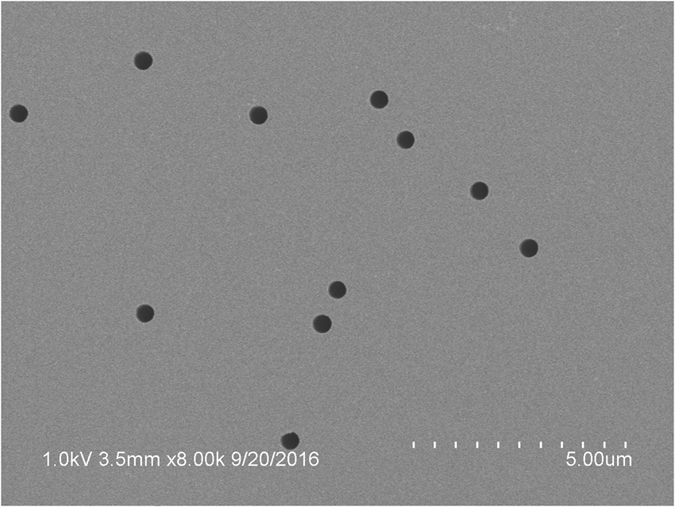
Scanning electron microscopy image of membrane surface on the side of large aperture of pores. Small apertures size is below the resolution.

Membranes with multiple conical nanopores are prepared similarly to ref. 26 by heavy-ion irradiation (Kr^28+^, 10.4 MeV, fluence 10^7^ cm^−2^, performed at GANIL, France) of *L* = 6 *μ*m thick polyimide foils (Kapton HN) and single-side chemical etching of ion-tracks. The etching was performed at 50 °C using a two chambers conductivity cell, one filled with NaOCl-15% etching solution, while the other with 1 M KI neutralizing solution. Transmembrane voltage of +1 V (with respect to the grounded neutralizing compartment) is applied to detect the breakthrough and help the neutralization of reactant as soon as the opening is achieved. Doing so, conical nanopores are obtained with a narrow size distribution, large opening radius *B* ≃ 200 nm (measured by scanning electron microscopy see Fig. 2) and small opening radius *b* ≃ 2 nm (estimated from the relation between this size, the conductivity of the electrolyte, and the measured conductance^26^).

Ionic current was measured using a Biologic-SP200 potentiostat with two Ag/AgCl electrodes. After etching, the two compartments of the conductivity cell were filled with the same electrolyte solution. In order to ensure that the electrode voltage corresponds to the actual transmembrane voltage, the area of the membrane was reduced down to 12.6 mm^2^ (i.e. 1.26 × 10^6^ pores) allowing its impedance to be larger than 95% of the total (see in Fig. 4 the current drop due to the membrane). Cyclic voltammetry was performed by applying a ±0.5 V sinusoidal voltage (small opening of pores at the grounded compartment) and measuring the current. Cycling was repeated until a stationary behavior (typically 2 or 3 periods). Only these last cycles are shown and discussed in the following. Reversibility was checked after each sample by measuring the membrane behavior with a reference electrolyte (acetic acid 0.1 M).

## Results

**Figure 3:**
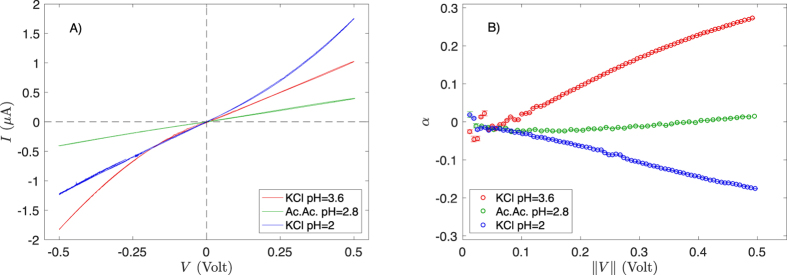
Cyclic voltammetry for conical nanopores filled with KCl 0.1 M solutions at pH = 3.6 and 2, respectively and acetic acid solution (c = 0.1 M, pH = 2.8). (**A**) Current *I* vs. voltage *V* measured at 1/32 Hz. (**B**) Rectification efficiency *α* vs. absolute value of voltage ||*V*|| (Eq. 3).

Dealing with entropic effects on ions transport, the major issue comes from static electric charges on pore-wall that may also lead to rectification of ionic current. Actually, track-etching of polyimide film produces ionized chemical groups at the surface of pore-wall: carboxylic acids and amines. Thus depending on pH, the net charge of pore-wall can be reversed and so the corresponding potential for free-ions^14^. In Fig. 3A, the ionic current *I* measured through polyimide conical nanopores is plotted as a function of the membrane voltage *V* for simple ions (i.e. non-polymeric) solutions at three different pH (KCl 0.1 M in acetate buffer at pH = 2 and 3.6, respectively, and acid acetic 0.1 M pH = 2.8). At pH = 2, carboxylic acids and amines are protonated resulting in a positive net charge of pore-wall, while at pH = 3.6 a sufficient quantity of carboxylic acids are dissociated to allow the inversion of the net charge. This is evidenced by a change of orientation of current rectification (inversion of the concavity of *I* vs. *V*). For acetic acid 0.1 M (pH = 2.8), no rectification is observed. These differences are better underlined by using the “normalized rectification efficiency”^19^,^27^:


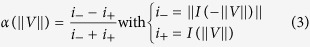


For a resistor *α* = 0, whereas for a diode *α* = ±1 depending on its direction. Results for *α* measured for simple ions solutions are plotted in Fig. 3B. We found that at pH = 2.8 ± 0.1, rectification is almost negligible for simple ions solutions. This pH is the isoelectric point of membranes. Results reported below were obtained at this pH, which is naturally obtained with polyacrylic acid (PAA) at a monomer concentration *c* = 0.1 M, because its dissociation constant (*pK*_*a*_ ≃ 4.5^28^) is close to the one of acetic acid. Note that at this concentration only 1.8% of acid groups are dissociated. This is the reason of the low level of the current (typically 1 *μ*A for ≈10^6^ pores, i.e. 1 pA per pore) and justifies the use of multipore-membranes for this study.

**Figure 4:**
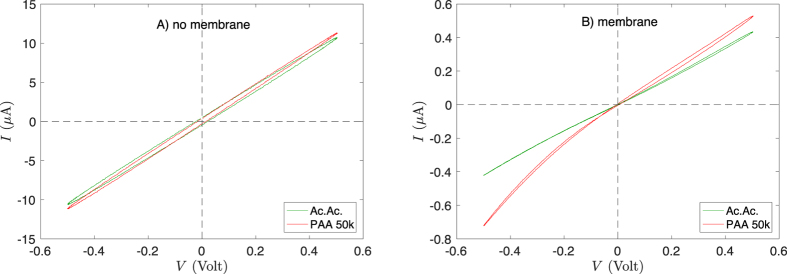
Cyclic voltammetry at 1 Hz for two electrolytes at pH = 2.8 ± 0.1: acetic acid at concentration *c* = 0.1 M (Ac.Ac.); polyacrylic acid, M = 50 × 10^3^ g/mol (PAA 50k) at monomer concentration *c* = 0.1 M. (**A**) No membrane between electrodes (**B**) membrane with conical nanopores between electrodes.

**Figure 5:**
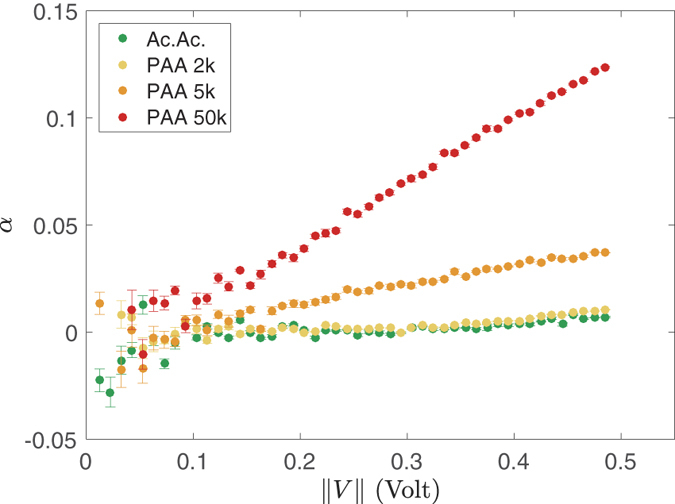
Cyclic voltammetry at 1 Hz: rectification efficiency *α* vs. absolute value of voltage ||*V*|| (Eq. 3) for acetic acid (Ac.Ac., *c* = 0.1 M) and PAA (𝑀 = 2, 5, and 50 kg/mol, respectively; monomer concentration *c* = 0.1 M) measured with membrane at the isoelectric point (pH = 2.8 ± 0.1).

Figure 4 shows cyclic voltammetry at 1 Hz for PAA with molecular weight *M* = 50 × 10^3^ g/mol and acetic acid at the same concentration. Introducing a membrane with conical nanopores between electrodes, causes a current drop but also a patent rectification with PAA as electrolyte, even though the membrane is at its isoelectric point. In Fig. 5, the rectification efficiency *α*, is plotted as a function of ||*V*|| for three different PAA molecular weights *M* and compared to acetic acid. For the two highest molecular weights, the *α*-values are clearly non-zero and comparable to the values obtained by simulations on other systems^19^,^27^. To appreciate this point, one has to keep in mind that for an entropic rectifier of ionic current, the contributions of anions and cations to the conductance add, so that ||*α*|| < 1 even if the entropic ratchet works perfectly. For instance, point-like ions contributing as a resistor and big counter-ions (with same mobility) contributing as a perfect diode would lead to ||*α*|| = 0.5. So, here the size dependence of the rectification efficiency is clear. This is our key result.

Examination of *I* = *f*(*V*) curves (see Figs 3A and 4B), shows that they tend to be linear for large enough ||*V*|| values. Hence, *α*(||*V*||) tends to a maximum *α*_*m*_ that can be estimated by:


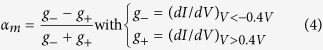


**Figure 6:**
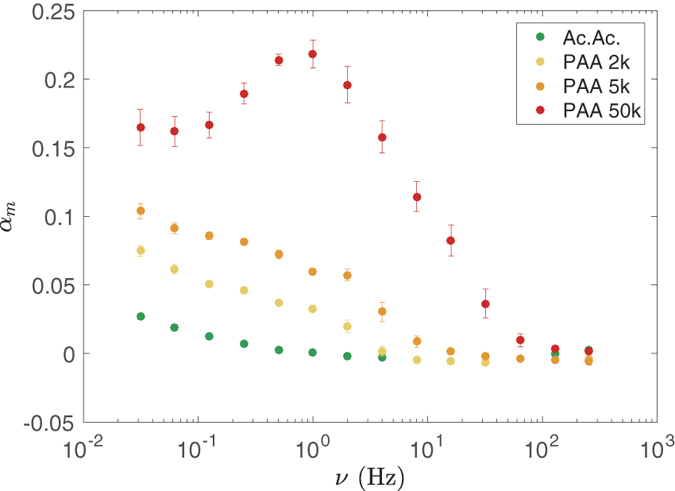
Maximum rectification efficiency *α*_*m*_ (Eq. 4) vs. cycle frequency *ν* for acetic acid (Ac.Ac. *c* = 0.1 M) and PAA of different molecular weights (𝑀 = 2, 5, and 50 kg/mol, respectively; monomer concentration *c* = 0.1 M) measured with membrane at the isoelectric point (pH = 2.8 ± 0.1).

The ratio *α*_*m*_ is plotted versus cycle frequency in Fig. 6 for acetic and PAA. We observe that *α*_*m*_ globally decreases for increasing frequency, but also passes by a smooth maximum for the highest molecular weight anion. Also, spectra are shifted to higher frequency for increasing anion size.

## Discussion

Let us estimate the different characteristic times of our experimental system. The first to consider is the transverse equilibration time that can be identified as the one needed for one chain to diffuse over the large aperture *B* of nanopore:


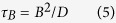


This time has to be compared to the time *τ*_*L*_ needed to cross the channel. Due to the non-constant cross-section area *A*(*x*) = *πr*(*x*)^2^ with *r*(*x*) = (*B* − *b*)/*L* × *x* + *b*, the electric field also is non-constant and writes


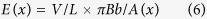


For one chain experiencing this field the bias strength is *f* = *E*(*x*)*γNe*, with *e* the elementary charge, *γ* the dissociation ratio of acid groups and *N* the number of statistical segments per chain (∝ molecular weight). In the stationary stage, this force is balanced by the friction *v*(*x*)*kT*/*D*, with *v*(*x*) the chain velocity, hence


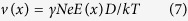


Thus the time *τ*_*L*_ to cross the entire pore writes:


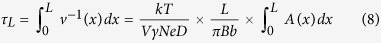


**Figure 7:**
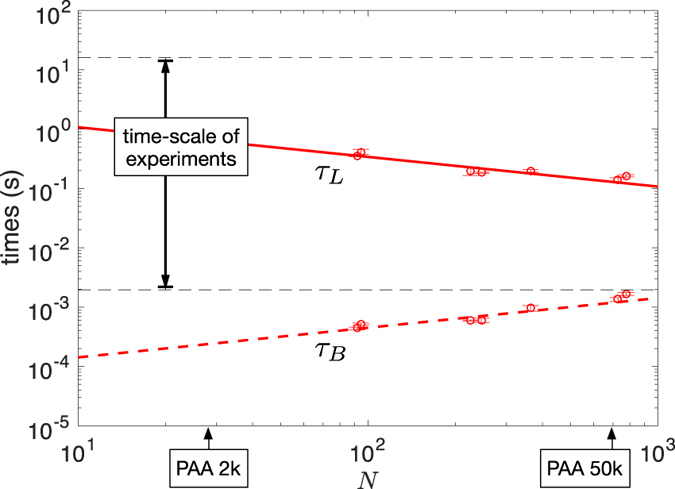
Characteristic times of PAA in our conical nanopores vs. number 𝑁 of segments per chain. *τ*_*B*_ (dashed red line) is the time needed for a chain to explore radially the pore (Eq. 5). *τ*_*L*_ (full red line) is the time needed to cross the membrane for a voltage of 0.5 V (Eq. 8). Data points correspond to *τ*_*B*_ and *τ*_*L*_ calculated from data of ref. 28. Horizontal dashed lines indicate the time-scale (frequency range) of Fig. 6.

The last integral is the volume of the entire pore. From measurements reported in literature^28^, one gets that at low pH the diffusion coefficient *D* of PAA scales as *D* = *D*_0_ × *N*^−1/2^, with *D*_0_ ≃ 10^−9^ m^2^/s. Note that *τ*_*B*_ increases for increasing chain length, whereas *τ*_*L*_ decreases. In Fig. 7, *τ*_*B*_ and *τ*_*L*_ were computed from experimental *D* values^28^ for *V* = 0.5 V, *L* = 6 *μ*m, *γ* ≃ 2 × 10^−2^ (i.e. pK_*a*_ = 4.5 and *c* = 0.1 M), *B* = 200 nm and *b* = 2 nm and plotted as a function of *N*. One can see that in our experiments *τ*_*B*_ is always at least 100 times smaller than *τ*_*L*_. This gives ground for the 1D treatment at the base of Eq. 2.

For cycle frequency *ν* higher than 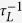, polyanions do not have time to cross the entire nanopore and remain localized in a small slice. Hence, 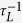 would give a simple estimate of the cycle-frequency above which rectification vanishes^27^. Recent simulations of polyelectrolyte chains translocating through asymmetric nanochannels confort this idea^29^. This may account for the shift of spectra to high frequency for increasing polyanions size (Fig. 6). Further attempt to rescale data of Fig. 6 by using *ντ*_*L*_ as reduced variable is at risk, because we still lack the y-axis reduced variable that should be likely necessary. However, it is quite remarkable that the drop of rectification efficiency is found to lie in the time window calculated for *τ*_*L*_. This point plainly argues that the energy potential experienced by polyanions extends over the full length *L* of nanopores and consequently supports the idea of its entropic origin.

Although the membrane is at the isoelectric point, let us consider the only well identified alternative origin of current rectification that should be an electrostatic potential due to the pore-wall. Electrostatic interactions are screened above the Debye’s length *λ*_*D*_ ≃ 7 nm for *c*_ions_ = *γc* = 2 × 10^−3^ M. So, the asymmetric electrostatic potential due to the pore-wall does not extend over the entire pore length but only over the length *l* = *λ*_*D*_ × *L*/(*B* − *b*) ≃ 200 nm from the cone tip. As a consequence, in Eq. 8 the volume to consider is 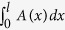, which is about (*L*/*l*)^3^ = 2 × 10^4^ times smaller than the volume of the entire pore, and also the time needed to cross this small region. This time is out of the time-window of our experiments and could not account for the shift of spectra reported in Fig. 6.

For the three PAA molecular weights here studied, the hydrodynamic radii *R*_*h*_ = *kT*/(6*πηD*), with *η* the viscosity of water, can be computed from the results of ref. 28. They are found to be equal to 1.2, 1.9 and 6 nm, respectively. As regards the pore size (small aperture radius *b* ≃ 2 nm), the question of chain blockade has to be examined. Actually, the electric field gradient due to the conical shape of pores is responsible for a stretching force applied on chains: *f*_*s*_ = *γNe* × ∇*E*(*x*) × *R*, with *R* the radius of gyration of chains. This causes chains to elongate longitudinally but also to shrink laterally. In this latter direction, the radius of chain can be estimated as 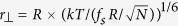 (see ref. 21 part I.4). Taking *R* ≃ *R*_*h*_, one gets the shrinking ratio 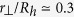 at the cone-tip (maximum field gradient) for the largest PAA chains, which should be sufficient to avoid chain blockades. Note that considering chain deformation does not weaken the previous calculation for *τ*_*B*_ and *τ*_*L*_ because deformation is only significant at the cone-tip and the time spent in this region is very short (the field scales as 1/*x*^2^ and is maximum at the tip, see Eq. 6) compared to those spent in the rest of the nano-channel, thus this time has a negligible weight in the expression of *τ*_*L*_ (Eq. 8). Also, note that this argument holds for any phenomenon that could occur at the cone-tip: providing that it does not drastically increase the time spent at the cone-tip (chain blockade) it has practically no incidence on *τ*_*L*_ and consequently on the measured current.

Further analysis of our results in relation with theory is not straightforward, especially the non-monotonic rectification spectrum in Fig. 6 that remains quite puzzling. In the literature, equation of motion through entropic barriers was not solved analytically, but with brownian dynamic simulations^20^,^24^,^25^,^27^. These simulations amount to integrate Langevin equation in some particular cases, which inevitably do not match to our experimental system. In particular, a difference is the coupling between the bias strength and particle size. Until now the case *f* ∝ *R* has been considered, whereas *f* ∝ *R*^2^ should be more suitable to fit to our case. May this work stimulates researches in this direction.

Nevertheless, in this paper we have shown that significant rectification of ionic current can be obtained with electrically neutral conical nanopores in case anions and cations widely differ in size. Our observations depend on ions size and cycle frequency. Currently, the entropic ratchet mechanism is the only able to account for them. Also, the entropic ratchet mechanism is possibly relevant for the understanding of facilitated or jammed-like transport observed for ionic-liquids confined in conical nanopores^26^, with likely outcomes for the improvement of electric batteries and cells that use confined geometries^30^.

## Additional Information

**How to cite this article**: Lairez, D. *et al*. The concept of entropic rectifier facing experiments. *Sci. Rep.* **6**, 38966; doi: 10.1038/srep38966 (2016).

**Publisher's note:** Springer Nature remains neutral with regard to jurisdictional claims in published maps and institutional affiliations.

## Figures and Tables

**Figure 1:**
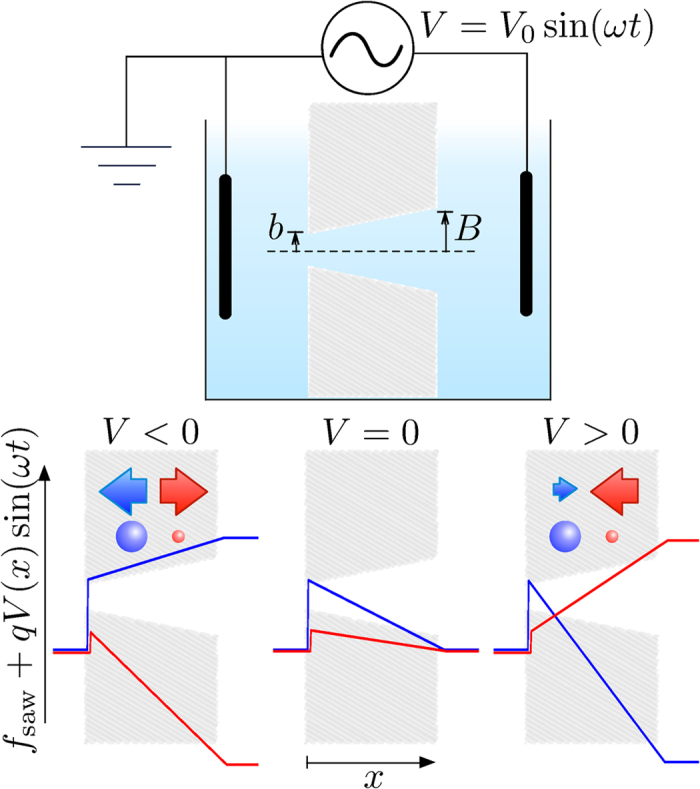
Conical nanopore as entropic rectifier: without transmembrane voltage, the sawtooth free energy potential *f*_saw_ is repulsive for both large anions (blue) and small cations (red), but smaller for the latter. For *V* > 0, the energy barrier for anions at the pore entrance (tip of cone) is higher than for cations when *V* < 0. One gets an ionic current diode. In case anions and cations have the same size, the entropic barriers have the same height and no rectification is expected. Here, *V*(*x*) stands for the variation of the electric potential on *x*. For a conical nanopore, this variation is not linear (the electric field is not constant, see Discusion Eq. 6). *f*_saw_ does not vary either linearly with *x*. In this schema, the linear behaviour of the sum of these two contributions is assumed for the sake of simplifying the drawing.
